# Frailty and postoperative outcomes in brain tumor patients: a systematic review subdivided by tumor etiology

**DOI:** 10.1007/s11060-023-04416-1

**Published:** 2023-08-25

**Authors:** Hanya M. Qureshi, Joanna K. Tabor, Kiley Pickens, Haoyi Lei, Sagar Vasandani, Muhammad I. Jalal, Shaurey Vetsa, Aladine Elsamadicy, Neelan Marianayagam, Brianna C. Theriault, Robert K. Fulbright, Ruihan Qin, Jiarui Yan, Lan Jin, Joseph O’Brien, Saul F. Morales-Valero, Jennifer Moliterno

**Affiliations:** 1 Department of Neurological Surgery, University of Massachusetts Medical School, Worcester, MA USA; 2 Department of Neurosurgery, Yale University School of Medicine, New Haven, CT USA; 3 The Chênevert Family Brain Tumor Center, Smilow Cancer Hospital, New Haven, CT USA; 4 Yale School of Public Health, New Haven, CT USA

**Keywords:** Meningioma, Frailty, KPS, Glioblastoma, Outcomes

## Abstract

**Purpose:**

Frailty has gained prominence in neurosurgical oncology, with more studies exploring its relationship to postoperative outcomes in brain tumor patients. As this body of literature continues to grow, concisely reviewing recent developments in the field is necessary. Here we provide a systematic review of frailty in brain tumor patients subdivided by tumor type, incorporating both modern frailty indices and traditional Karnofsky Performance Status (KPS) metrics.

**Methods:**

Systematic literature review was performed using PRISMA guidelines. PubMed and Google Scholar were queried for articles related to frailty, KPS, and brain tumor outcomes. Only articles describing novel associations between frailty or KPS and primary intracranial tumors were included.

**Results:**

After exclusion criteria, systematic review yielded 52 publications. Amongst malignant lesions, 16 studies focused on glioblastoma. Amongst benign tumors, 13 focused on meningiomas, and 6 focused on vestibular schwannomas. Seventeen studies grouped all brain tumor patients together. Seven studies incorporated both frailty indices and KPS into their analyses. Studies correlated frailty with various postoperative outcomes, including complications and mortality.

**Conclusion:**

Our review identified several patterns of overall postsurgical outcomes reporting for patients with brain tumors and frailty. To date, reviews of frailty in patients with brain tumors have been largely limited to certain frailty indices, analyzing all patients together regardless of lesion etiology. Although this technique is beneficial in providing a general overview of frailty’s use for brain tumor patients, given each tumor pathology has its own unique etiology, this combined approach potentially neglects key nuances governing frailty’s use and prognostic value.

## Introduction

An age-related syndrome of physiologic decline, frailty refers to an overall decreased state of health characterized by diminished reserves and resistance to stressors linked with adverse health outcomes [1]. In 2010 two seminal studies linked frailty to preoperative risk factors, postoperative outcomes, and complications for certain groups of surgical patients [2, 3]. Studies over the next several years continued analyzing frailty in different patient populations; however, lack of standardized frailty metrics made drawing comparisons between studies difficult. In response, several indices emerged, including the 11-point modified frailty index (mFI) [4], 5-point mFI [5], Johns Hopkins Adjusted Clinical Groups (JHACG), and Hopkins Frailty Score (HFS) [6], with recent studies identifying clinically relevant connections between frailty and patients with brain tumors [4–7]. Given Karnofsky Performance Status (KPS) scores have historically been influential in assessing neurosurgical oncology patients’ functional status, other studies have utilized KPS to define frailty [8].

In the first major systematic review of frailty in brain tumor patients, Huq et al. note that relative to mFI, fewer studies employed the JHACG or HFS. Patients classified as frail by the JHACG or HFS were associated with higher mortality, complications, reoperation rates, length of stay (LOS), charges, costs, discharge disposition, and readmission rates [6, 9]. Given most frailty measures include a combination of history, physical examination, and determination of physical capabilities, the mFI-11 and subsequent mFI-5 were developed [4, 5]. Huq et al. highlight that most studies employing mFI-5 categorized patients into one of three frailty levels based on raw scores or percentages of the total frailty items [6]. Some studies treated mFI-5 as a dichotomous variable, while others treated it as a continuous variable [6, 10–12]. Studies utilizing the mFI-11 also placed patients into one of several categories based on integer or fractionated scores. The decision to group patients into two (frail vs. non-frail), three (non-frail vs. moderately frail vs. highly frail), or four (non-frail vs. low frailty vs. intermediately frail vs. highly frail) categories varied by study and cohort demographics [6]. Ultimately, Huq et al. note both the mFI-5 and mFI-11 have been associated with mortality, complications, LOS, charges, discharge disposition, and readmission rates amongst brain tumor patients [6]. The mFI-5 has been linked with operation time [13], and the mFI-11 has been linked with end-of-life care needs and reoperation rates [14].

Still, in reviewing various tumor types together, Huq et al. [6] potentially neglect key nuances governing frailty indices’ inherent value. Their combined approach, while crucial to developing an overview, may underestimate frailty’s use and prognostic value given each tumor pathology has a unique etiology. As the body of literature exploring neurosurgical outcomes in brain tumor patients continues to grow, we aim to capture some of these nuances by presenting the first review of frailty in brain tumor patients subdivided by tumor type, incorporating both modern frailty indices and traditional KPS metrics. Benign and malignant lesions will be reviewed separately, with a major focus placed on glioblastoma and meningioma given their relatively higher prevalence amongst all tumor types [15].

## Methods

A systematic literature review was performed using PRISMA guidelines. PubMed and Google Scholar were queried for articles related to frailty, KPS, and brain tumor outcomes. The keywords “frailty” and “KPS” were used in combination with “glioma,” “glioblastoma,” “meningioma,” “acoustic neuroma,” and “vestibular schwannoma.” Searches were performed combining the term “frailty” with each of the aforementioned tumor types. To maximize output, a search was also done combining the terms “frailty” and “brain tumor.” Two authors (HQ, KP) screened abstracts and reviewed papers for study inclusion. A third author (JT) served as a reviewer/arbitrator to achieve consensus. Only articles that described novel associations between frailty or KPS and primary intracranial tumors were included. Studies examining frailty, including relevant review articles and meta-analyses, were included regardless of their frailty criteria; however, any studies that failed to define explicit frailty criteria were excluded. Those focusing on metastatic disease or radiotherapy were also generally excluded. Only studies written in English were included. References in the selected articles were reviewed to obtain a more holistic, longitudinal description of frailty in brain tumor patients.

## Results

**Fig. 1:**
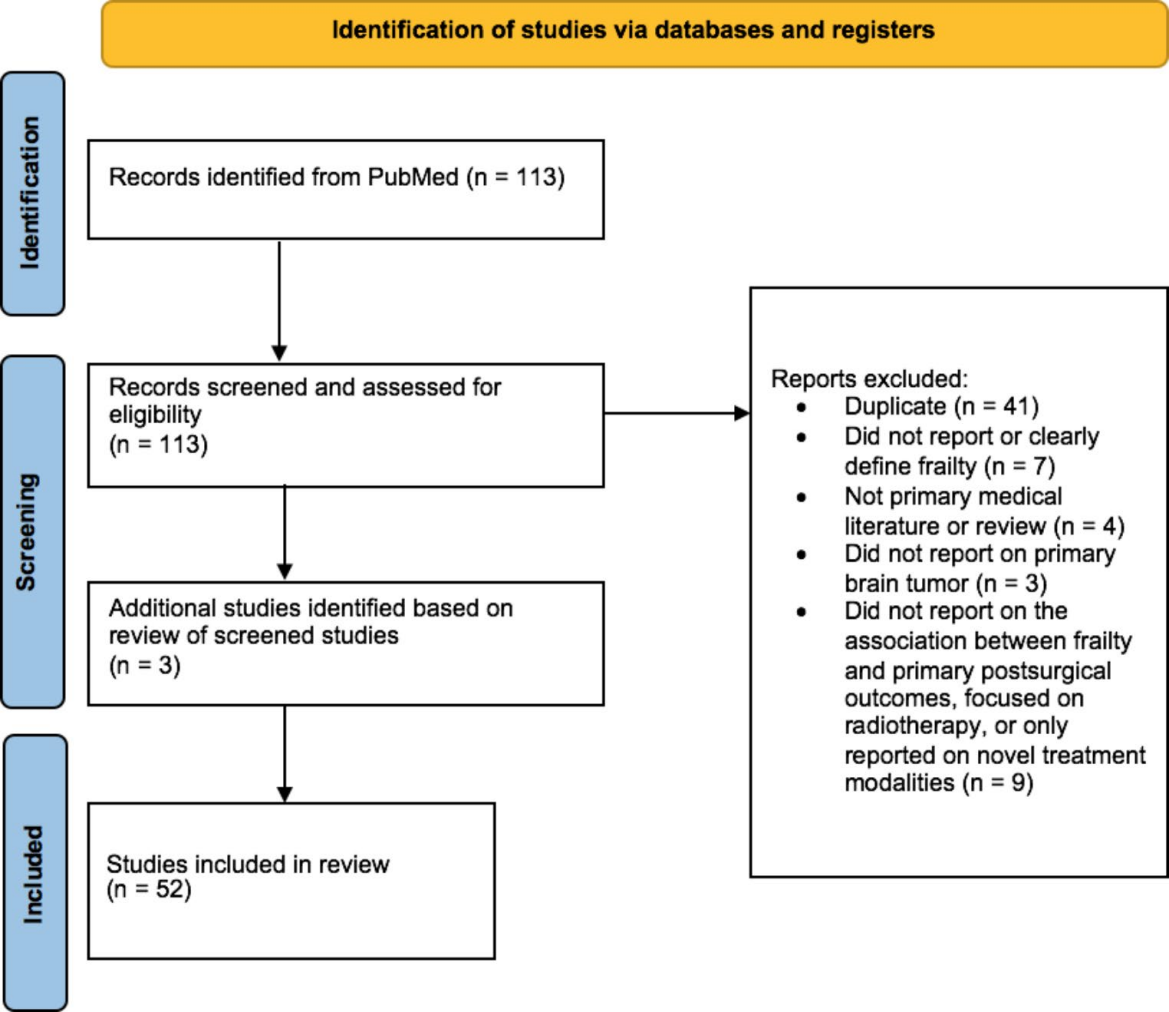
Preferred Reporting Items for Systematic Reviews and Meta-Analyses (PRISMA) flow diagram. Adapted from: Page MJ, McKenzie JE, Bossuyt PM, Boutron I, Hoffmann TC, Mulrow CD, et al. The PRISMA 2020 statement: an updated guideline for reporting systematic reviews. BMJ 2021; 372:n71. 10.1136/bmj.n71. [70]

Our initial search yielded 113 citations. After 41 duplicates were removed, 7 did not report on or clearly define frailty, 4 were not primary medical literature or review studies, 3 did not report on brain tumor pathologies, and 8 did not report on associations between frailty and primary postoperative outcomes, focused only on radiotherapy outcomes, or only reported on a single novel treatment modality. Once these exclusion criteria were applied and additional references were reviewed for potential inclusion, systematic review of frailty in brain tumor patients yielded 52 publications encompassing 294,373 patients (Fig. 1; Table 1). Retrospective institutional studies were the most common, followed by retrospective national database studies, reviews, and prospective institutional studies respectively (Table 1). Nearly all studies were large cohort studies.

Amongst malignant lesions, 16 studies focused on GBM [14, 16–30]. Amongst benign tumors, 13 focused on meningiomas [31–43], and 6 focused on vestibular schwannomas [13, 44–48]. 17 grouped all brain tumor patients together [6, 9, 10, 49–62]. Relatively few studies employed frailty indices other than the mFI-5, mFI-11, and JHACG [38, 56, 63]. Some studies using the mFI tended to group patients into one of 3–4 frailty categories using cutoff scores, while others treated frailty as a binary variable when correlating with outcomes [6, 13, 14, 22, 44, 45, 48]. The most standardization in defining frailty was observed in studies addressing vestibular schwannoma followed by meningioma and GBM, respectively. Seven studies incorporated both frailty indices and KPS into their analyses, with multiple reporting KPS and frailty as predictors of postoperative outcomes [16, 19, 22, 29, 30, 56, 64]. However, lack of KPS score standardization posed a major limitation. One study noted frailty scales were more sensitive, identifying more vulnerable patients than KPS alone [56]. Another reported that KPS improvement after tumor resection did not always predict outcomes in comparison to frailty indices [29].

GBM studies reported significant relationships between frailty and overall survival, extended hospital LOS, hospital readmission and associated complication rates, need for post-discharge specialist care, need for extra-familial aid post-discharge, and postoperative complications [14, 16, 17, 19–26, 29, 30]. Meningioma studies similarly found significant relationships between frailty and mortality, extended hospital LOS, hospital readmission rates, and postoperative complications [32–42]. However, studies also noted a relationship between frailty and unplanned reoperation, non-home discharge dispositions, KPS deterioration [32, 35, 36]. A relationship between frailty and a variety of postoperative complications, extended hospital LOS and readmission rates, non-home discharges, and mortality was reported in patients with vestibular schwannoma [44–48, 65].

## Discussion

### Frailty across brain tumor types

Studies analyzing multiple tumor pathologies together have largely focused on understanding frailty’s predictive value, finding that higher frailty was significantly associated with increased risk for non-home discharge [9, 62], postoperative complications [9, 60–62], extended length of ICU and overall hospital stays [9, 52, 60–62], mortality [54, 55, 60, 62], readmission rates [54], all-payer hospital costs [61], and decreased rates of postoperative day one discharge [10]. Although Bonney et al. report frail patients were not more likely to be readmitted than their non-frail counterparts following brain tumor resection, this may be influenced by the authors’ decision to use the JHACG frailty index as opposed to the more established mFI [49]. While Torres-Perez et al. report frailty scales identified vulnerable patients across tumor types with greater reliability than KPS alone, they note not every frailty scale significantly corresponded with postoperative outcomes [56]. These findings highlight the importance of using more established standardized frailty indices to prognosticate outcomes.

Huq et al. used the mFI to explore postoperative complications across all patients with brain tumors on a more granular level, reporting higher rates of pulmonary embolism, physiological/metabolic derangement, respiratory failure, and sepsis per each mFI-5 point increase [58]. While such combined studies are beneficial in obtaining an overview of frailty’s applicability to all brain tumor patients, dividing literature by tumor subtype can offer more nuanced perspectives on frailty’s relevance across pathologies and demographic groups.

### Frailty in patients with glioblastoma

Most literature concerning frailty and GBM has focused on the elderly, who tend to have poor prognoses with limited responses to treatment [66]. Though Bruno et al. acknowledged age may factor into more aggressive tumor biology, they highlighted that elderly patients with poor clinical statuses due to comorbidities may not be able to tolerate surgery or adjuvant chemotherapy, making physicians more reluctant to offer aggressive treatments, in turn contributing to worse prognoses for this demographic [66]. Lorimer et al.’s cross-sectional survey of UK based consultant neuro-oncologists further supports this theory [67]. Another study of geriatric patients who underwent craniotomy for lobar GBM revealed that frailer patients were not only less likely to undergo surgical resection than their less frail counterparts, but also experience increased hospital stays, an increased overall risk of complications, and decreased overall survival (OS) [17]. Frailty was quantified using the mFI and was associated with these outcomes independent of age, KPS, cardiovascular risk, and comorbid disease [17]. At least four other studies also explored frailty’s relationship to postoperative outcomes in elderly patients with GBM [19, 24, 26, 29]. Although each used different metrics to define frailty, all four found significant negative relationships between frailty and OS. In fact, Krezlin et al. reported that KPS improvement postoperatively did not reliably predict postoperative outcomes in patients with GBM unless they were already frail, suggesting that clinical and comorbidity consideration alongside KPS is critical to identifying patients for aggressive treatment and allowing for accurate prognostication [29].

Still, treatment pathways for geriatric patients with GBM remain controversial. While some propose that treatment may accelerate frailty progression in the elderly [18, 68], others argue that frailty or low KPS should not hinder a patient’s treatment [21, 28]. In fact, Wick et al. state that while the decision to pursue chemoradiotherapy can be influenced by KPS, chemotherapy should not be withheld even from patients with low KPS [28]. Our own institutional study examining patients with GBM also supports this idea, demonstrating that elderly patients with relatively low preoperative KPS scores can still show significant improvement postoperatively [69].

Beyond the geriatric patient demographic, two major studies have explored frailty’s relationship to postoperative outcomes in patients with GBM more generally. In a retrospective review, Botros et al. reported an increased odds of 30-day readmission with each 10-point decrease in KPS score and with each single-point increase in mFI-5 scores [16]. Readmitted patients were also noted to have lower mean KPS scores relative to their non-readmitted counterparts [16]. In another review, Klingenschmid et al. reported on pre and postoperative frailty using KPS and the Clinical Frailty Scale (CFS) [22, 63]. Both preoperative and postoperative KPS and CFS scores correlated, suggesting that the CFS may be equally reliable to KPS for patients with GBM [22]. Higher scores on both scales not only significantly correlated with decreased OS, but also decreased survival by roughly the same percent per scale point [22]. While age and KPS scores both predicted OS, the two variables only marginally correlated with each other, suggesting there may be distinct multiple frameworks to approach frailty [22]. Still, Katiyar et al. argue that the mFI-11 is a better predictor than age of certain surgical outcomes, including hospital and ICU LOS, postoperative complications, in-hospital mortality, and psychosocial/financial difficulty post-discharge [14].

Ultimately, while studies have connected increased frailty in patients with GBM to malnutrition [50], poor follow-up care [23], and decreased temporal muscle thickness [25, 27], many studies define frailty using incomparable metrics. This overall lack of standardization combined with GBM’s progressive nature may be contributing to disputes regarding frailty’s role in guiding care for geriatric patients with malignant primary brain tumors. Although only three studies have explored frailty’s relationship to postoperative outcomes outside the geriatric demographic, frailty’s relationship to OS, increased LOS, and readmissions is clear [14, 22, 30].

### Frailty in patients with meningioma

Relative to patients with GBM, more studies have explored frailty’s relationship to postoperative outcomes and clinical characteristics for patients with meningioma. Various measures of frailty have been deemed predictive of survival and morbidity amongst patients with meningioma [43]. Many studies have also incorporated younger demographics, potentially enabling more meaningful longitudinal comparisons between studies across age groups.

In a study of elderly patients with primarily low-grade, skull-base meningiomas, age, sex, KPS, tumor size, tumor location, and frailty (defined as a combination of body mass index and serum albumin levels) were evaluated as risk factors for postoperative deterioration over the course of one year [38]. Preoperative KPS scores, body mass index (BMI), and low serum albumin levels were each linked with poor prognostic factors [38]. The BMI component was identified as a risk factor for KPS score deterioration in the immediate postoperative period [38], suggesting alternative frailty frameworks may offer more nuanced assessments of postoperative functional status than preoperative KPS scores can predict. Isobe et al. offered further insights, highlighting that both tumor location and serum albumin measures of frailty were risk factors for KPS score deterioration at discharge [35]. These variables along with age and tumor size were significant risk factors for perioperative intracranial complications [35].

Other studies have approached frailty longitudinally, directly comparing the relative importance of specific clinical variables on outcomes in older vs. younger patient populations. Two out of three major frailty studies in meningioma suggest that differences in outcomes are unrelated to tumor biology or characteristics, arising instead from natural differences in KPS secondary to aging and medical comorbidities [31, 39]. While declining functional status in older patients predicted perioperative complications, the overall perioperative complication profile of older and younger patient demographics following resection were similar [39].

In contrast, Ikawa et al. found frailty predicted operative outcomes for younger patients with meningioma [34]. Unlike previous studies, they utilized the mFI-5, applying it to a larger cohort of 8138 database patients, finding that mFI-5 ≥ 2 was a more significant risk factor than chronological age for poor outcomes, including mortality and complication rates, in patients under 65 [34]. Their results highlight a need to examine frailty in meningioma more critically across age groups.

Another study defined early postoperative deterioration in meningioma patients as a 20-point or more drop in KPS [42]. Unfavorable long-term functional autonomy and quality of life was defined as a postoperative KPS decrease of at least 20 points and overall quality of life below the 75th percentile of the examined population [42]. Using a 34-point Frailty Index score, Tariciotti et al. demonstrated that preoperative frailty directly predicted both early postoperative deterioration, and long-term unfavorable outcomes along with other key tumor characteristics [42]. However, they were unable to report whether this relationship is longstanding because of inherent frailty or from known longstanding medical comorbidities.

Still, studies indicate that higher mFI scores are independently associated with overall postoperative morbidity and mortality. Institutional and large database studies alike have linked mFI with non-routine discharge disposition, extended hospital LOS, readmission, and postoperative complication development, including life-threatening complications and mortality for patients with meningioma [32, 33, 37, 41, 42]. Like those reporting on GBM and frailty, many of these studies in patients with meningioma treated frailty as a binary variable, using various cutoff scores to distinguish frail vs. non-frail patients. However, there does appear to be more standardization in the indices utilized by studies analyzing frailty in patients with meningioma than in patients with GBM.

### Frailty in patients with other types of brain tumors

Though limited, several studies have explored frailty’s relationship with postoperative outcomes in patients with vestibular schwannoma. Like studies in patients with meningioma and GBM, Nasrollahi et al. report that frail geriatric patients with vestibular schwannoma are more likely to experience increased readmission rates, LOS, and non-home discharges [47]. The authors also report on higher postoperative infection, facial paralysis, urinary tract infection, hydrocephalus, and dysphagia rates specifically in geriatric patients with vestibular schwannoma [47]. While Helal et al. suggest frail, elderly patients can safely undergo surgery for vestibular schwannoma, their small cohort was a limiting factor [46].

Overall, frailty studies in vestibular schwannoma demonstrate relatively more methodological standardization, with the vast majority utilizing the mFI-5 or mFI-11 to classify frailty [13, 44, 45, 48]. Patients were classified into one of three groups (non-frail, intermediate frailty, and frail) with raw mFI scores of 2 or 3 serving as the high frailty cutoff score. Both Casazza et al. [44] and Goshtasbi et al. [13] note that patients with higher mFI scores tended to have longer hospital LOS than those with lower scores. They also indicate that frailty bears little relationship with increased complication rates. In contrast, a large database study of patients with vestibular schwannoma suggests that increasing frailty is strongly associated with development of postoperative hemorrhagic or ischemic stroke and increased LOS, particularly amongst non-White patient demographics [45]. While both Dicpinigaitis et al. [45] and Casazza et al. [44] utilize the mFI-11, they do utilize different cutoff scores to classify highly frail patients, potentially contributing to the differences reported by each. Ultimately, Tang et al. note that while mFI is significantly associated with perioperative outcomes, a customized frailty index for patients with vestibular schwannoma outperforms any mFI in predicting routine discharge [48].

## Conclusion

Our comprehensive, systematic literature review identified several patterns of overall postsurgical outcomes reporting for patients with brain tumors. Amongst patients with GBM, frailty is associated with OS, extended hospital LOS, hospital readmission, complications, and post-discharge specialist or extra-familial care. Studies of patients with vestibular schwannoma and meningiomas reported relationships between frailty and postoperative complications, extended hospital LOS, readmission rates, non-home discharges, and mortality. A few meningioma-specific studies also reported relationships between frailty, unexpected reoperation, and KPS deterioration. While we report frailty is a more significant risk factor than age for poor outcomes in patients with meningioma, similar conclusions cannot be drawn about patients with other tumor types without further studies in younger patient demographics. In the future, it could be beneficial to use a tumor pathology-specific frailty index to incorporate clinical and functional status history alongside tumor genomics and radiographic features to better enable shared-decision making and long-term prognostication for patients with brain tumors.

**Table 1:** Studies Identified in Systematic Literature Review

Authors & Year	Study Type	Primary Instrument of Frailty Assessment	Tumor Pathologies Included	Number of Patients
Armocida et al., 2022[31]	Retrospective, Institutional	KPS	Meningioma	340
Bonney et al., 2021[49]	Retrospective, National Database	JHACG	Multiple	87,835
Botros et al., 2022[16]	Retrospective, Institutional	mFI-5, KPS	GBM	69
Casazza et al., 2020[44]	Retrospective, Institutional	mFI-11	Vestibular Schwannoma	218
Cloney et al., 2016[17]	Retrospective, Institutional	mFI-11	GBM	243
Cohen-Inbar, 2019[43]	Review		Meningioma	
Cohen-Inbar, 2019[18]	Review		GBM	
Cole et al., 2022[32]	Retrospective, National Database	mFI-5	Meningioma	5818
Dicpinigaitis et al., 2021[45]	Retrospective, Multi-Institutional and National Database	mFI-11	Vestibular Schwannoma	27,313
Dicpinigaitis et al., 2021[33]	Retrospective, National Database	mFI-11	Meningioma	20,250
Giaccherini et al., 2019[19]	Retrospective, Institutional	KPS, multiple others including non-mFI CSHA-based frailty index	GBM	34
Goshtabi et al., 2020[13]	Retrospective, National Database		Vestibular Schwannoma	1405
Harland et al., 2020[9]	Prospective, Institutional	HFS	Multiple	260
Helal et al., 2021[46]	Retrospective, Institutional	mFI-5	Vestibular Schwannoma	24
Henry et al., 2021[60]	Retrospective, National Database	mFI-5	Multiple	17,912
Huq et al., 2021[20]	Retrospective, Institutional	mFI-5, nutritional status	GBM	242
Huq et al., 2020[58]	Retrospective, Institutional	mFI-5	Multiple	1692
Huq et al., 2021[50]	Retrospective, Institutional	mFI-5	Multiple	2325
Huq et al., 2022[6]	Review		Multiple	
Ikawa et al., 2022[34]	Retrospective, National Database	mFI-5	Meningioma	8138
Isobe et al., 2018[35]	Retrospective, Institutional	KPS	Meningioma	265
Jimenez et al., 2022[36]	Retrospective, Institutional	mFI-5	Meningioma	396
Jimenez et al., 2022[51]	Retrospective, Institutional	mFI-5	Multiple	2519
Jimenez et al., 2020[37]	Retrospective, Institutional	mFI-5	Meningioma	154
Jimenez et al., 2021[52]	Retrospective, Institutional	mFI-5	Multiple	654
Katiyar et al., 2020[14]	Retrospective, Institutional	mFI-11	GBM	276
Khalafallah et al., 2020[10]	Retrospective, Institutional	mFI-5, mFI-11	Multiple	1692
Klingenschmid et al., 2022[21]	Retrospective, Institutional	KPS, CFS	GBM	121
Klingenschmid et al., 2022[22]	Retrospective, Institutional	KPS, CFS	GBM	289
Kolakshyapati et al., 2018[38]	Retrospective, Institutional	KPS, BMI, albumin	Meningioma	57
Krenzlin et al., 2021[29]	Retrospective, Institutional	KPS, Gronigen Frailty Index	GBM	104
Mirpuri et al., 2022[23]	Retrospective, Institutional	mFI-5	GBM	244
Mungngam et al., 2022[53]	Prospective, Institutional	Frailty Instrument of the Survey of Health, Aging, and Retirement in Europe	Multiple	85
Nair et al., 2022[30]	Retrospective, Institutional	KPS, mFI-5	GBM	265
Nasrollahi et al., 2022[47]	Retrospective, National Database	JHACG	Vestibular Schwannoma	396
Pryzbylowski et al., 2022[39]	Retrospective, Institutional	KPS, age	Meningioma	287
Rahmani et al., 2020[24]	Retrospective, National Database	Multiple, including BMI, dependent functional status, weight	GBM	1016
Richardson et al., 2019[59]	Retrospective, Institutional	mFI-11	Multiple	424
Roux et al., 2022[40]	Retrospective, Institutional	mFI-11, Charlson Comorbidity Index	Meningioma	102
Sadhwani et al., 2022[25]	Review and meta-analysis	Temporalis muscle thickness	GBM	3283
Sastry et al., 2020[54]	Retrospective, National Database	mFI-5	Multiple	20,333
Schneider et al., 2020[26]	Retrospective, Institutional	mFI-11	GBM	110
Shahrestani et al., 2020[61]	Retrospective, National Database	JHACG	Multiple	13,342
Tang et al., 2022[48]	Retrospective, National Database	mFI-11, Charlson Comorbidity Index	Vestibular Schwannoma	32,465
Tariciotti et al., 2022[42]	Retrospective, Institutional	Multiple, including 34-item Frailty Index and Charlson comorbidity index	Meningioma	165
Ten Cate et al., 2022[27]	Retrospective, Institutional	Temporalis muscle thickness	GBM	245
Theriault et al., 2020[41]	Retrospective, Institutional	mFI-11	Meningioma	76
Thommen et al., 2022[55]	Retrospective, National Database	mFI-5, Elevated Risk Analysis index	Multiple	30,951
Torres-Perez et al., 2021[56]	Prospective, Institutional	KPS, FRAIL scale questionnaire incorporating multiple frailty indices and phenotypes	Multiple	131
Wang et al., 2021[57]	Retrospective, Institutional	mFI-11	Multiple	659
Wick et al., 2018[28]	Review		GBM	
Youngerman et al., 2018[62]	Retrospective, National Database	mFI-11	Multiple	9149
